# Walk test and school performance in mouth-breathing children

**DOI:** 10.5935/1808-8694.20130037

**Published:** 2015-11-02

**Authors:** Ana Paula Dias Vilas Boas, Fernando Augusto de Lima Marson, Maria Angela Gonçalves de Oliveira Ribeiro, Eulália Sakano, Patricia Blau Margosian Conti, Adyléia Dalbo Contrera Toro, José Dirceu Ribeiro

**Affiliations:** a Specialist in Pediatric Physical Therapy - FCM, Unicamp. MSc student in Children and Adolescents' Healthcare - School of Medical Sciences (FCM), Unicamp; b MSc in Children and Adolescents' Healthcare. PhD student in Children and Adolescents' Healthcare - Department of Pediatrics. Researcher - Department of Medical Genetcs; c PhD in Children and Adolescents' Healthcare - FCM, Unicamp. Research Coordinator of the Pulmonary Physiology Lab; d PhD in Medical Sciences/Otorhinolaryngology - FCM (UNICAMP). Professor of Otorhinolaryngology UNICAMP; e MSc in Children and Adolescents' Healthcare - FCM, Unicamp (Physical Therapist - FCM, Unicamp; f PhD in Children and Adolescents' Healthcare. Professor - Department of Pediatrics - FCM, Unicamp; g Full Professor - Department of Pediatrics - Unicamp. Researcher and Professor - Department of Pediatrics - Unicamp. Campinas State University - School of Medical Sciences - FCM, Department of Pediatrics

**Keywords:** exercise tolerance, mouth breathing, nasal obstruction, performance tests

## Abstract

**Abstract:**

In recent decades, many studies on mouth breathing (MB) have been published; however, little is known about many aspects of this syndrome, including severity, impact on physical and academic performances.

**Objective:**

Compare the physical performance in a six minutes walk test (6MWT) and the academic performance of MB and nasal-breathing (NB) children and adolescents.

**Method:**

This is a descriptive, cross-sectional, and prospective study with MB and NB children submitted to the 6MWT and scholar performance assessment.

**Results:**

We included 156 children, 87 girls (60 NB and 27 MB) and 69 boys (44 NB and 25 MB). Variables were analyzed during the 6MWT: heart rate (HR), respiratory rate, oxygen saturation, distance walked in six minutes and modified Borg scale. All the variables studied were statistically different between groups NB and MB, with the exception of school performance and HR in 6MWT.

**Conclusion:**

MB affects physical performance and not the academic performance, we noticed a changed pattern in the 6MWT in the MB group. Since the MBs in our study were classified as non-severe, other studies comparing the academic performance variables and 6MWT are needed to better understand the process of physical and academic performances in MB children.

## INTRODUCTION

Breathing is a vital function, closely dependent on proper nasal patency^1^. Mouth breathing (MB) happens due to a switch from nasal breathing (NB) into the mouth breathing (MB), lasting for more than 6 months. MB is caused by mechanical events, allergic and non-allergic inflammatory diseases, congenital malformations and tumors^1^, ^2^, ^3^.

MB consequences may stem from: (i) changes to physiological mechanisms: especially due to local inflammation and allergy; and (ii) facial changes: especially due to congenital anatomical changes. As a consequence of nasal obstruction, there may be reduction in olfactory stimuli, increase in pulmonary hyperresponsiveness, stuffed nose, dry lips, sleepiness, lack of attention, snoring, low physical strength, obstructive sleep apnea syndrome (OSAS), open mouth, crowding teeth, long face, dropped eyes, deep dark circles under the eyes, postural changes, nasal itching and behavioral vices (nervous twitches)^1^, ^3^, ^4^, ^5^, ^6^, ^7^. Upper airway obstructions in the first childhood, depending on severity and duration may cause MB due to inflammation of the vocal folds, pharyngeal and palatine tonsils^1^, ^4^.

There are controversies as to the definition and diagnosis of MB, since mouth-breathing individuals may breathe through their noses in varied degrees, and others, despite breathing through their mouths, may not have anatomical obstruction of their noses. It is paramount to make the etiological diagnosis of MB, especially as to the presence of allergic rhinitis and pharyngeal and palatine tonsil hyperplasia^2^.

In recent decades, there has been a great interest in understanding the ethological and pathophysiological mechanisms involved in MB, with numerous papers published in the national and international scientific literature. On the other hand, very little is known about numerous aspects associated with this syndrome, including its levels of severity and repercussions on athletic and academic performance.

With the knowledge acquired by our group from previous studies^1^, ^2^, we tried to study the athletic and academic performance of mouth-breathing children and adolescents; considering the consequences which may arise from MB, analyzing its influence on functional and learning capacities. Thus, our goal has been to assess athletic performance using the six minute walk test (6MWT) and school performance in a group of MB children, compared to a group of NB children.

## METHOD

We carried out a cross-sectional, descriptive, prospective study, with a control group, made up of mouth-breathing (MB) and nasal-breathing (NB) children and adolescents, with ages varying between 7 and 11 years. The study was carried out at UNASP (Adventist University Center of São Paulo) Hortolândia campus - IASP with the Basic Education I students, between March of 2010 and April of 2011.

The children from both genders had their MB diagnoses carried out by means of a clinical assessment, rhinos-copy, oroscopy and otoscopy^1^, ^2^, ^4^; and MB was defined as a replacement of the nasal pattern for the mouth-breathing pattern, for a period equal to or longer than six months. MB diagnosis was carried out by otorhinolaryngologists and physical therapists of the State University of Campinas (Unicamp), based on a joint analysis of the assessment model proposed by Abreu et al.^7^. The control group encompassed children of the same age, students of the same school, of the same school class, who did not have MB clinical signs or complaints.

We measured their height (Staturometer - *Personal Caprice Sanny* ®, São Paulo, São Bernardo, Brazil) and weight (ID1500® Filizola Scale São Paulo, São Paulo, Brazil). The body mass index (BMI) was calculated utilizing the weight/height^2^ (kg/m^2^) formula and, posteriorly, compared to the *Central Disease Control* (2000) for BMI, at the cutting points: malnutrition (< percentile 5); adequate (> percentile 5 and ≤ percentile 85); overweight (> percentile 85 and ≤ percentile 95) and obesity (> percentile 95)^8^.

**Figure 1:**
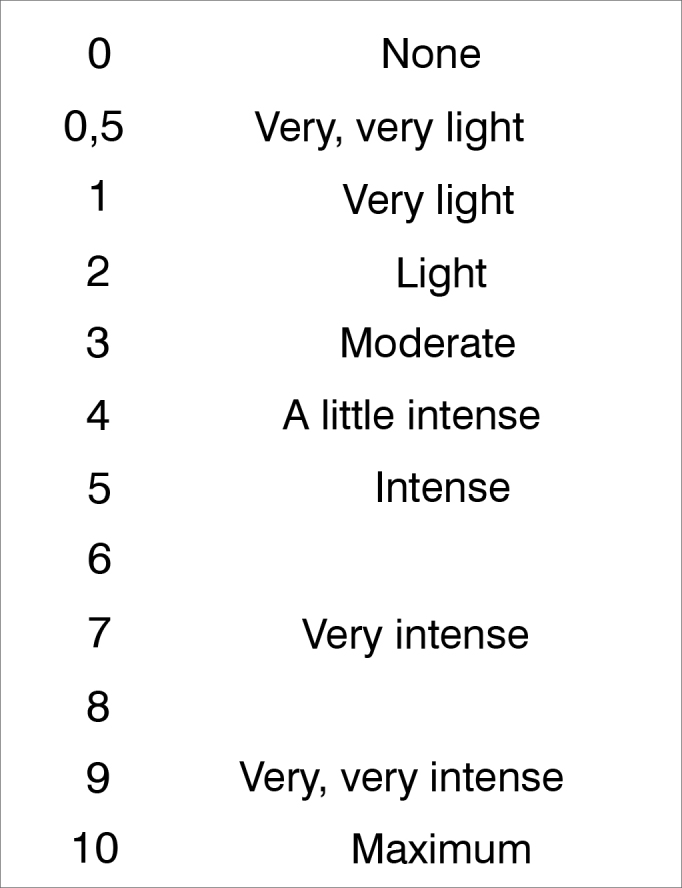
Modified Borg Scale. Levels of dyspnea^10^.

The 6MWT was carried out according to the standards from the American Thoracic Society (ATS)^9^. We initially asked each individual to remain seated for 5 minutes in order to collect rest variables. We checked respiratory rate (RR) - measured during one minute, when we observed abdominal and thoracic movements, with a hand on the abdomen to confirm the beginning and end of each respiratory cycle, periphery oxygen saturation (SpO_2_) and heart rate (HR) - measured by the portable pulse oximeter (*Heal Force Prince* 100, Shanghai, China) and the modified Borg scale (MBS)^9^, ^10^ (Figure 1). The cardiorespiratory variables were checked at two time intervals along the 6MWT: during rest and at the sixth minute, both in the first and in the second tests. Results were compared between the tests and along the time points.

The children were educated about the 6MWT by means of a previous demonstration. Every minute we told the child about the remaining time for the end of the test and we verbally encouraged the children to continue (“You are doing great”; “Come on” or “Keep going”). After the sixth minutes, the individual was instructed to stop where he/she was, and we measured the distance from the cone all the way to the point the child had stopped, we also measured the RR (respiratory rate), HR, SpO_2_ and the MBS with the individual seating down.

Since the ATS does not establish the minimum time interval for the 6MWT repetition which is not intended for training, we establish a 15-minute rest time. Following that, the same individual was asked to perform the second 6MWT^2^, ^9^, ^10^. The 6MWT was carried out with one individual at a time, and should the individual feel any discomfort, the test was stopped^9^.

School performance was assessed by the summation of the report card grades, obtained during the school year of 2010, divided by the total number of courses taken.

This study was approved by the Ethics in Research Committee of the School of Medical Sciences (FCM)/UNICAMP (#1066/2009) and all the parents/guardians signed the Informed Consent Form.

### Statistical Analysis

The sample power calculation was carried out by the G*Power 3.1.2 software, with a mean sample size effect for the test carried out with the two groups (MB and NB); α= 0.05 and β= 0.80; the sample size was 143 individuals.

In the statistical analysis we used the *SAS System for Windows* version 9.2 (SAS Institute Inc, 2002-2008, Cary, NC, USA), *Microcal Origin*, version 5.0 (*Microcal Software Inc*, 1991-1997, Northampton, MA, USA) and SPSS vs 17.0 (*Statistical Package for Social Science - SPSS*, Armonk, New York, USA) software packages.

In order to compare proportions, we used the Chi-square test. To compare the continuous or ordering measures or between tests along time, we used the ANOVA variance analysis for repetitive measures with transformation by posts. For multiple comparison purposes, we employed the profile test by contrasts. In comparing the MB and NB groups as far as distance walked is concerned, we used the *t-Student* test by data distribution. The level of significance adopted for the statistical tests was 5%^11^, ^12^, ^13^.

## RESULTS

Our study included 156 children, 104 NB (66.67%) and 52 MB (33.33%). All MB children were classified as not severe (i.e. did not require specialized medical treatment according to the family). The gender distribution between NB and MB groups was similar. MB incidence was higher among boys - 25 (36.23%) - when compared to girls - 27 (31.03%), with no statistically significant difference (*p* = 0.494). The frequency of obesity (BMI ≥ percentile 95) was high in the two groups analyzed - 37 (23.72%) -, without statistically significant differences between the groups (*p* = 0.105). In the NB group, among the 104 patients, 30 (28.85%) were obese, with BMI ≥ P95; while of the 52 patients in the MB group, seven (15.56%) were obese, with BMI ≥ P95.

The following variables: age, grade point average, weight and height were statistically similar for both genders, considering the two groups: NB and MB (*p* > 0.05).

**Figure 2:**
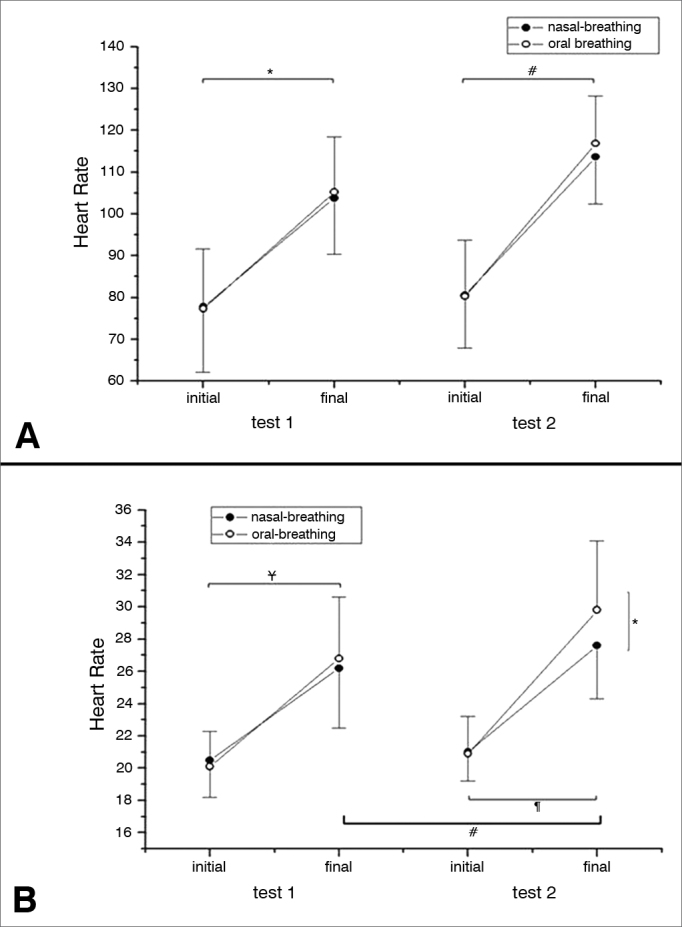
Cardiac and respiratory rates. A. HR in the different groups analyzed (NB and MB) during the first and second 6MWT carried out. For each 6MWT, we have the HR initial and final values. There were no significant differences between the analyzed groups (NB and MB), nor between the tests 1 and 2. HR was higher after the six-minute walk in the first test (* *p* < 0.0001) and in the second test (^#^*p* < 0.0001) regardless if the group was NB or MB. The significance level adopted for the analysis was 0.05. B. RR in the different groups analyzed (NB and MB) during the first and second 6MWT carried out. For each 6MWT we describe the RR initial and final values. In the second test, the MB group had higher RR than the NB (* *p* = 0.0011). The MB in the second 6MWT held in comparison to the first, had a higher RR (^#^*p* = 0.0038). In the comparison between the beginning and the end of the 6MWT, regardless of the NB and MB groups, in both tests there was a higher value for the RR (test 1: ^¥^*p* = 0.0011 and test 2: ¶*p* = 0.0011). The level of significance adopted for the analysis was 0.05. HR: Heart rate; RR: Respiratory rate; MB: Mouth-breathing; NB: Nasal-breathing; SpO_2_: Oxygen peripheral saturation; 6MWT: 6-minute walk test.

**Figure 3:**
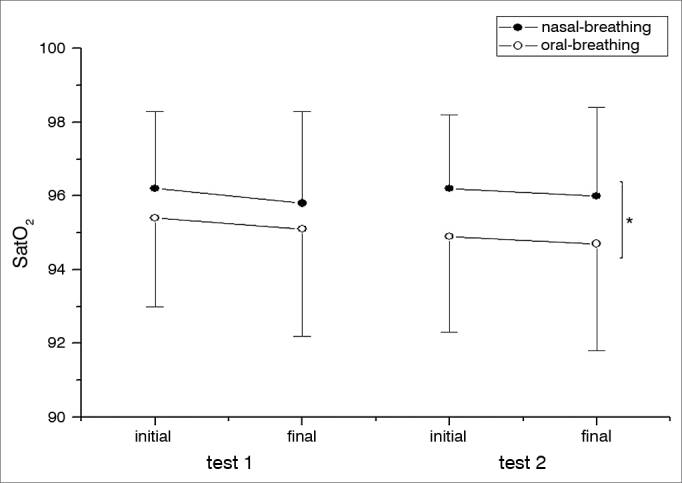
Oxygen peripherical saturation. SpO_2_ in the different groups analyzed (B and MB) during the first and second 6MWT. For each 6MWT we describe the SpO_2_ initial and final values. In the second test, the SpO2 was higher in the NB group when compared to MBs. * *p* = 0.0175. The significance level adopted for the analysis was 0.05. MB: Mouth-breathing; NB: Nasal-breathing; SpO_2_: Oxygen peripheral saturation; 6MWT: 6-minute walk test.

**Figure 4:**
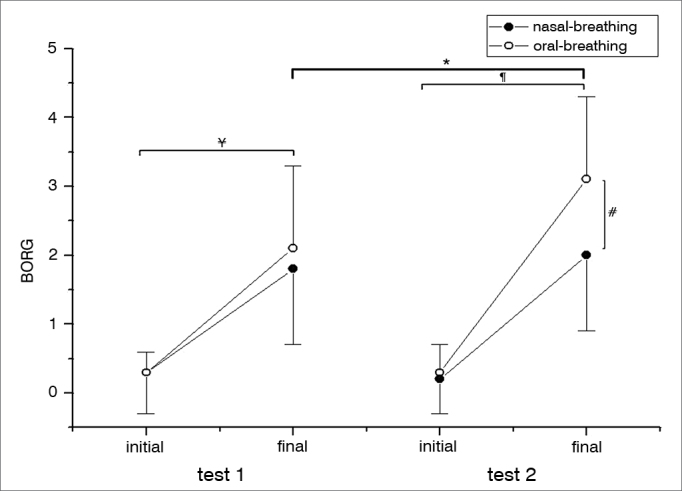
Comparison with the Modified Borg Scale. The MBS in the different groups analyzed (NB and MB) during the first and the second 6MWT. For each 6MWT we describe the initial and final values of the MBS. In comparing tests 1 and 2 in the NB group, the MBS value was higher in test 2 (* *p* = 0.0005). In test 2, the MB group had the higher MBS when compared to the NB individuals at the end of the 6MWT (^#^*p* = 0.0088). Regardless of the group analyzed (NB or MB), there was an increase in the MBS value in test 1 (^¥^*p* = 0.0005) and test 2 (^¶^*p* = 0.0005). The level of significance adopted for the analyses was 0.05. MB: Mouth-breathing; NB: Nasal-breathing; MBS: Modified Borg Scale; 6MWT: 6-minute walk test.

Although the HR was higher after the end of the test in both groups studied, there was no statistically significant difference before and after the 6MWT between MB and NB (*p* = 0.4632) (Figure 2A). The RR was different between the first and the second 6MWT for MB individuals; while in the second 6MWT there was a difference in RR for MB individuals, a higher value (Figure 2B). SpO_2_ values were lower for the MB group when compared to the NB at the second 6MWT (Figure 3). There was a statistically significant difference among the MB children between the end of the first and the second 6MWT, as far as the MBS is concerned (Figure 4).

**Figure 5:**
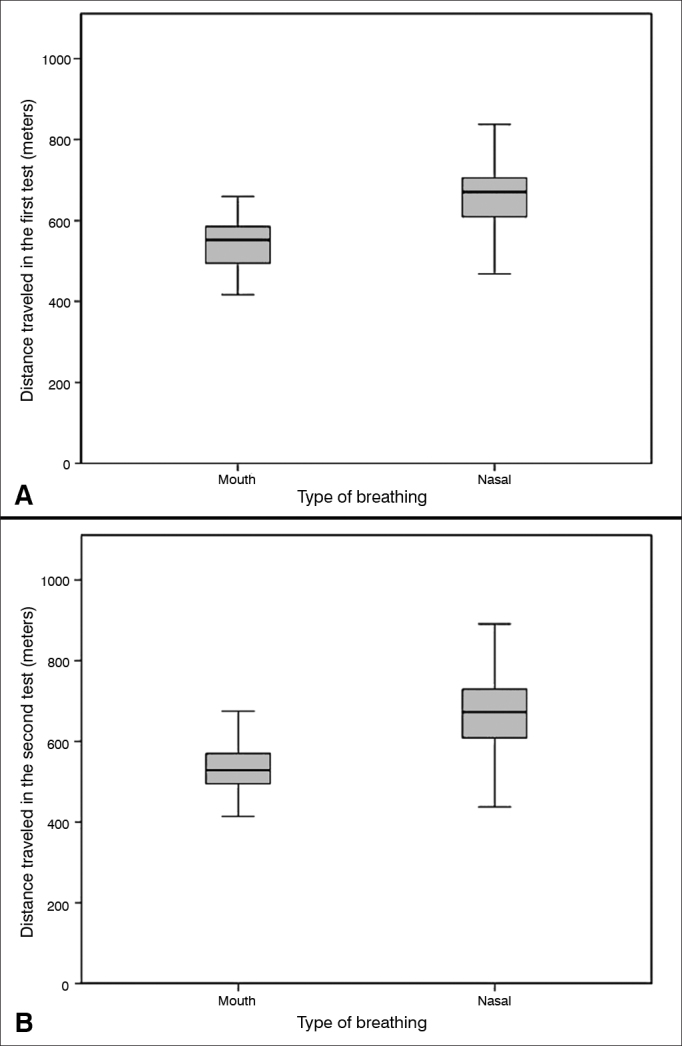
Analysis of the distance traveled in six minutes in the walk test A: Distance traveled in the first 6MWT, comparing the MB group (550.67 ± 72.22) with the NB group (667.14 ± 88.97). *Student-t* test. The level of significance adopted for the analysis was 0.05, (T = -7.902), *p* < 0.0001). B: Distance traveled in the second 6MWT, comparing the MB group (543.40 ± 79.40) with the NB group (667.13 ± 90.67). *Student-t* test. The level of significance adopted for the analyses was 0.05. MB: Mouth-breathing; NB: Nasal-breathing; 6MWT: 6-minute walk test.

All the individuals completed the 6MWT without the need for interruption. The mean values of the 6MWT in the first test was, respectively, 550.67 m (±72.221 m) and 667.14 m (±88.979 m), for the MB group and NB (*p* < 0.001). In the second test, the mean value for the 6MWT was, 543.40 m (±79.406 m) and 667.13 m (±90.678 m) for the MB and NB groups, respectively (*p* < 0.001) (Figure 5).

The school performance analysis in relation to the 6MWT by the Student's t-test did not show differences between the MB (35.117 ± 2.506) and NB (35.502 ± 2.544) groups (*p* = 0.372). Just as in the Spearman's linear correlation test, MB first test (*p* = 0.624) and second test (*p* = 0.422) and NB in the first test (*p* = 0.935) and second test (*p* = 0.738). When we compared the percentages of NB and MB with the best performances, we did not find statistically significant differences between the groups (by the 35.705 median, the patients were broken down into two groups: (i) worst performance - school mean values lower than the median and (ii) better performance - school mean values higher than the median; 57.7% of the patients with MB and 46.2% with NB had the worst school performance) (*p* = 0.234).

## DISCUSSION

MB is frequently found in school-age children and adolescents in Brazil, having repercussions in the stomatognathic, muscle-skeletal and respiratory systems, and changes to body posture. Nevertheless, there is very little information on the influence of these changes in an individual's functional capacity and school performance^1^, ^2^, ^4^. In previous studies carried out in our clinic, we assessed the differences between NB and MB, with the use of the 6MWT in children with changes to their respiratory system^1^, ^2^, ^14^. We observed that MB may bring about respiratory and postural changes, and affect males more often. Prior studies have shown that MB impacts negatively on the biomechanics of breathing and capacity to exercise, anterior head tilt and acts as a mechanism of compensation to improve the respiratory function muscles. The 6MWT was sensitive and reproducible in children with respiratory system changes, and it is an instrument used to assess cardiorespiratory functional capacity^1^, ^2^, ^14^.

In the present study, as observed by other authors, there was a greater prevalence of MB among males. And, differently from girls, boys have upper airway anatomical structures of smaller gauge and greater prevalence of allergic rhinitis - which are the most frequent causes of MB^1^, ^2^, ^3^, ^5^.

Anthropometric variables (weight, height, age, gender and BMI) were similar in both groups, which speaks for sample homogeneity, although some studies have found a greater prevalence of anorexia and low weight and height gain in MB children. Other authors have reported weight gain in MB children after surgical removal of the adenoids^1^, ^6^, ^15^, ^16^. The children in this study went to a private school, with freedom to feed in the school cafeteria, where there is a high supply of caloric food - which may have contributed to the high obesity index in NB and MB children.

Considering the findings of respiratory mechanics reduction in MB, we deem it is necessary to investigate its repercussion in physical performance. The reduction in respiratory muscle power is triggered by the disorganization of the postural pattern, inadequate use of respiratory muscles and nasal reflex block, which control how deep breathing gets and airway gauge. These factors establish lower pulmonary volumes and capacities, impacting chest expansion and alveolar ventilation, causing a reduction in arterial oxygen pressure, reflecting on exercise tollerance^3^. Another hypothesis is the single airway theory, in such a way that MB may extend its effects to the pulmonary region and impact the physiological response to exercise. MB may trigger changes to the muscular, circulatory and respiratory systems, with repercussions in the individual's physiological mechanisms. The respiration mode, nasal or oral, mal also change airflow in the upper airways and influence the deposit of particles and gas absorption in the lungs^3^.

The 6MWT is a practical and inexpensive test, able to estimate the submaximal functional capacity in individuals with chronic diseases. It is one of the tests that best mimics daily life activities, being an alternative to maximum exercise tests which, despite being considered gold standard to estimate exercise tolerance, require high cost equipment, specialized personnel and they are difficult to perform^2^, ^9^, ^14^.

The ATS guideline for the 6MWT was created for use with the adult population. There are physical differences between adults and children, which are more complex than anthropometric measures. The physiological characteristics of children and adolescents are in constant change, their systems are under a stage of growth, development and maturation, under the influence of genetics and extrinsic factors: level of physical activity, body composition, nutritional status, socioeconomic status, gender, ethnics, climate and geographic location. Children have a different pattern of change to their bodies when compared to adults, which affects physical performance and may cause physiological changes during physical exercise^9^.

We have noticed that MB children have different values when compared to their NB counterparts, insofar as the 6MWT variables are concerned. The results confirm that the search for simple tests, such as the 6MWT, may be useful to characterize the cardiorespiratory condition of MB children and adolescents. And such fact was confirmed in another study^2^.

Although the 6MWT is an instrument utilized to calculate and standardize PD in distinct populations^17^, ^18^, ^19^, ^20^, to compare chronic patients with healthy ones^2^, ^14^, test the 6MWT intrinsic variables^14^, ^21^ and compare the 6MWT efficacy with other tests^22^, we only found one study which also assessed the HR, RR, SpO_2_ and MBS variables in MB and NB groups^2^.

According to the ATS, there are numerous factors which may affect the PD in the 6MWT in a negative way (low stature, shorter lower limbs, advanced age, high body weight, female gender, impaired cognition, 6MWT use in an inadequate space, cardiorespiratory and/or orthopedic disease) and, in a positive way (high height, male gender, great motivation, training carried out before the test, some medication and oxygen supplement)^9^.

In our study, the MB group showed 6PD values near the ones found by Okuro et al.^2^, whom used the same methodological approach. The comparison of our data with others in the literature is made difficult because most of the studies have the 6MWT with differences in the anthropometric characteristics (weight, height, gender, age and BMI), with normality curves sampling recruitment obtained from different populations. Some trials did not follow ATS's recommendations or used populations with other conditions than MB^14^, ^17^, ^20^, ^21^, ^22^.

In the literature, only Okuro et al. Used the 6MWT in MB and NB children; however, the MB sample was recruited from a clinic specialized in MB individuals, encompassing individuals with severe MB. In our sample, MB individuals were recruited from a school with non-severe MB ^2^.

We did not find changes in school performance between NB and MB individuals. We believe that this is due to the fact that we analyzed only the average grade of the school report card (information provided by the school), and we were unaware of the grade recovery criterion. Other studies assessing the learning in MB individuals used specific tests. As it happened in our study, the results were not statistically significant^23^.

The school performance results between NB and MB, in our study, may be associated with the characteristics of the population analyzed. MB children and adolescents did not have the severity characteristics of those described by Conti et al.^1^ and Okuro et al.^2^. In our trial, the children were selected in a school and there was no follow up in hospitals as far as their MB was concerned.

As a limitation in our study, we may include the lack of an assessment instrument for a more accurate school performance assessment and comparative analysis with severe MB individuals, which must be further studied in the future.

## CONCLUSION

MB children show a significant increase in RR; SaPO_2_ and MBS reduction in the second 6MWT test, when compared to the group of NB individuals. Other studies are needed to assess cardiorespiratory, physical and school performance changes in MB children.

## ACKNOWLEDGMENTS

We would like to thank the physicians and collaborators of the Pediatrics Ward and Pediatrics Department.

We would also like to thank the Laboratory of Molecular Genetics (www.laboratoriomultiusuario.com.br) for their help in statistical data analysis.

We would like to thank Professor Thatia Regina Bonfim for her support, and all the students, parents, employees and IASP managers.
